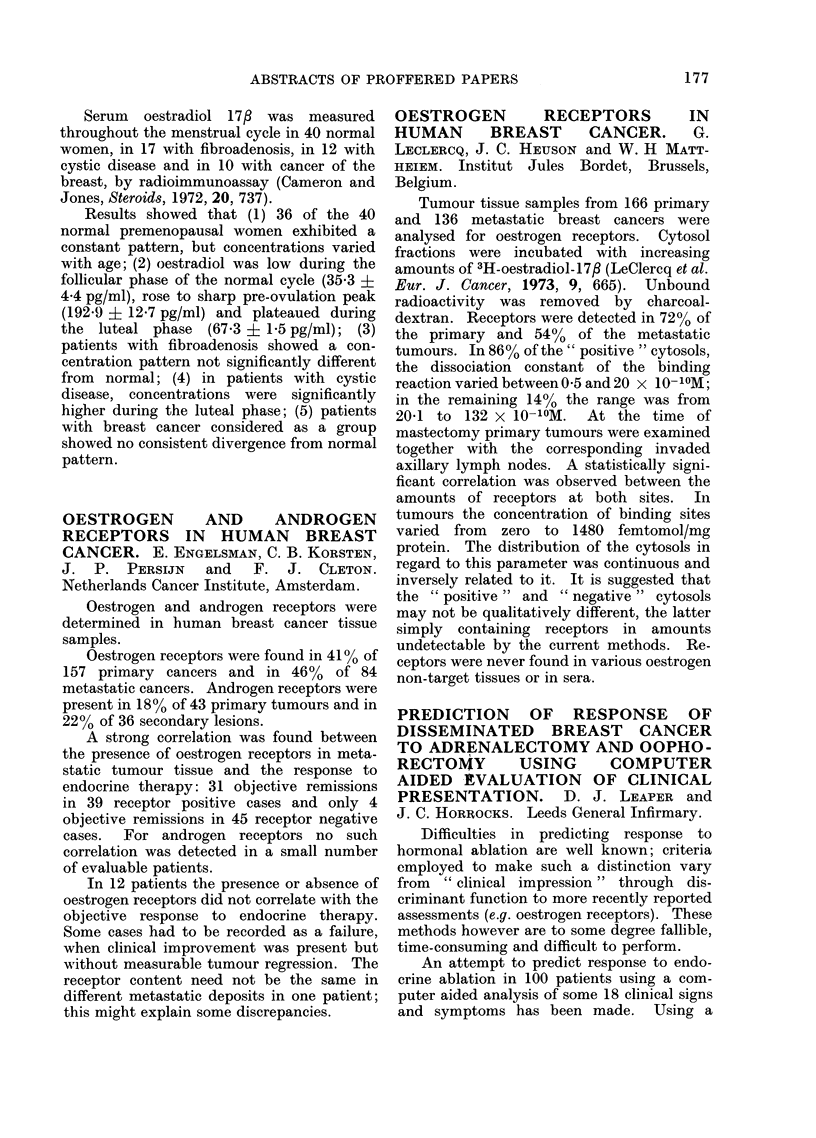# Proceedings: Oestrogen and androgen receptors in human breast cancer.

**DOI:** 10.1038/bjc.1974.145

**Published:** 1974-08

**Authors:** E. Engelsman, C. B. Korsten, J. P. Persijn, F. J. Cleton


**OESTROGEN AND ANDROGEN RECEPTORS IN HUMAN BREAST CANCER.** E. ENGELSMAN, C. B. KORSTEN, J. P. PERSIJN and F. J. CLETON. Netherlands Cancer Institute, Amsterdam.

Oestrogen and androgen receptors were determined in human breast cancer tissue samples.

Oestrogen receptors were found in 41% of 157 primary cancers and in 46% of 84 metastatic cancers. Androgen receptors were present in 18% of 43 primary tumours and in 22% of 36 secondary lesions.

A strong correlation was found between the presence of oestrogen receptors in metastatic tumour tissue and the response to endocrine therapy: 31 objective remissions in 39 receptor positive cases and only 4 objective remissions in 45 receptor negative cases. For androgen receptors no such correlation was detected in a small number of evaluable patients.

In 12 patients the presence or absence of oestrogen receptors did not correlate with the objective response to endocrine therapy. Some cases had to be recorded as a failure, when clinical improvement was present but without measurable tumour regression. The receptor content need not be the same in different metastatic deposits in one patient; this might explain some discrepancies.